# Production and covalent immobilisation of the recombinant bacterial carbonic anhydrase (SspCA) onto magnetic nanoparticles

**DOI:** 10.1080/14756366.2017.1316719

**Published:** 2017-05-12

**Authors:** Rosa Perfetto, Sonia Del Prete, Daniela Vullo, Giovanni Sansone, Carmela M.A. Barone, Mosè Rossi, Claudiu T. Supuran, Clemente Capasso

**Affiliations:** a Istituto di Bioscienze e Biorisorse, CNR, Napoli, Italy;; b Dipartimento Neurofarba, Sezione di Scienze Farmaceutiche, and Laboratorio di Chimica Bioinorganica, Polo Scientifico, Università degli Studi di Firenze, Sesto Fiorentino, Italy;; c Dipartimento di Biologia, Università degli Studi di Napoli, Federico II, Napoli, Italy;; d Dipartimento di Agraria, Università degli Studi di Napoli, Federico II, Portici, Napoli, Italy

**Keywords:** Carbonic anhydrase, metalloenzymes, magnetic nanoparticles, high cell density, immobilised enzyme, thermostability

## Abstract

Carbonic anhydrases (CAs; EC 4.2.1.1) are metalloenzymes with a pivotal potential role in the biomimetic CO_2_ capture process (CCP) because these biocatalysts catalyse the simple but physiologically crucial reaction of carbon dioxide hydration to bicarbonate and protons in all life kingdoms. The CAs are among the fastest known enzymes, with *k*_cat_ values of up to 10^6^ s^−1^ for some members of the superfamily, providing thus advantages when compared with other CCP methods, as they are specific for CO_2_. Thermostable CAs might be used in CCP technology because of their ability to perform catalysis in operatively hard conditions, typical of the industrial processes. Moreover, the improvement of the enzyme stability and its reuse are important for lowering the costs. These aspects can be overcome by immobilising the enzyme on a specific support. We report in this article that the recombinant thermostable SspCA (α-CA) from the thermophilic bacterium *Sulfurihydrogenibium yellowstonense* can been heterologously produced by a high-density fermentation of *Escherichia coli* cultures, and covalently immobilised onto the surface of magnetic Fe_3_O_4_ nanoparticles (MNP) via carbodiimide activation reactions. Our results demonstrate that using a benchtop bioprocess station and strategies for optimising the bacterial growth, it is possible to produce at low cost a large amount SspCA. Furthermore, the enzyme stability and storage greatly increased through the immobilisation, as SspCA bound to MNP could be recovered from the reaction mixture by simply using a magnet or an electromagnetic field, due to the strong ferromagnetic properties of Fe_3_O_4_.

## Introduction

Lately, a group of metalloenzymes named carbonic anhydrases (CAs; EC 4.2.1.1) has acquired a great importance in biomedical and biotechnological applications, such as in the achievement of artificial respiration systems, selective biosensors for metal ions, and post-combustion CO_2_ capture processes (CCPs)^1–5^. CCPs are acquiring a great importance, as CO_2_ emission reduction is becoming a pressing issue in the industrial production sectors. In fact, the increase of gases with greenhouse effect in the atmosphere, including CO_2_, coming from the combustion of fossil materials, represents one of the leading factors of environmental stress, and is considered a major cause of climate change^6–8^. The production of “eco-compatible” combustible materials and/or the reduction of CO_2_ accumulation in the atmosphere represent the highest priority for a better quality of human life. In the field of CCP, a number of CO_2_ sequestration methods have been proposed. Among them are noteworthy the sequestration of CO_2_ as a carbonate salt^9^, which is interesting since the carbonate minerals constitute the largest reserve of CO_2_ on earth, as well as the chemical absorption of CO_2_ by alkanolamines, considered as highly toxic substances^7^^,^^10^. In recent years, CAs have emerged as biocatalyst with a pivotal potential role in the biomimetic CCP. The reason for the enormous importance of CAs in CCP can be summarised as follow: (i) CAs are metalloenzymes present in all life kingdoms, and they catalyse the simple but physiologically crucial reaction of carbon dioxide hydration to bicarbonate and protons: CO_2_ + H_2_O⇄ HCO_3_^-^ + H^+^^11–13^; (ii) CAs are among the fastest known enzymes, with a *k*_cat_ value of up to 10^6^ s^−1^ for some members of the superfamily, which is almost 10 million times faster than the noncatalysed reaction^11^^,^^14^^,^^15^; (iii) the biomimetic approach based on the use of a CA has several advantages, when compared with the other CCP methods, as it is specific for CO_2_, being also an eco-compatible process; (iv) the CO_2_ solubilisation as ions (bicarbonate and carbonate) allows its further use, for example for the growth of algae or other microorganisms, as well as in a variety of industrial applications, in which calcium bicarbonate derived from the reaction of calcium salts/hydroxide with the carbonate ion produced by the hydratase reaction, is employed^16^^,^^17^.

In biology, the conversion of carbon dioxide to bicarbonate, and *vice versa*, is associated with processes such as respiration, photosynthesis, pH regulation and homeostasis of the organism, CO_2_ and HCO_3_^-^ transport, several biosynthetic processes, production of body fluids, bone resorption, and other physiological processes, mostly investigated in plants and mammals^13^^,^^18–20^. Moreover, CAs are essentials in the biomineralisation process in mollusks, corals and other marine organisms^19–27^ playing an important role during the calcium carbonate shell formation, acid-base regulation, calcification and mineralisation^28^^,^^29^. Thus, all life kingdoms need CAs to make faster the naturally reversible but slow CO_2_ hydration, due to the slow rate of carbonation reaction (10^−1 ^s^−1^). The CA superfamily includes seven distinct classes known as the α, β, γ, δ, ζ, η and θ-CAs^18^^,^^19^^,^^25^^,^^26^^,^^30–38^. Some of the catalytically active α- and θ-CAs can also catalyse the hydrolysis of esters, such as 4-nitrophenyl acetate (4-NpA)^39^. However, no esterase activity was detected so far for enzymes belonging to the other five classes (β-, γ-, δ-, ζ- and η-CAs)^40^. The α-, β-, δ-, η- and perhaps θ-CAs, use Zn(II) ions at the active site, the γ-CAs are probably Fe(II) enzymes (but they are active also with bound Zn(II) or Co(II) ions)^41–48^, whereas the ζ-class CAs are cambialistic enzymes, active both with Cd(II) or Zn(II) bound within the active site in order to perform the physiologic reaction catalysis^13^^,^^49^^,^^50^. The coordination chemistry of zinc in proteins involves N, O and S donors of the side chains of histidine, glutamate/aspartate and/or cysteine. The metal ion from the CA active site is coordinated by three His residues in the α-, γ-, δ- and probably the θ-classes, by one His, and two Cys residues in β- and ζ-CAs or by two His and one Gln residues in the η-class, with the fourth ligand being a water molecule/hydroxide ion acting as nucleophile in the catalytic cycle of the enzyme^15^^,^^20^^,^^24^^,^^25^^,^^51^^,^^52^. All CAs identified in animal systems belong to the α-class^14^^,^^53^. CAs identified in plants and algae belong to the α-, β-, γ-, δ- and θ-classes; fungi encode for α- and β-CAs; protozoa encode for α-, β- or η-CAs; bacteria encode for enzymes belonging to the α-, β- and γ-CA families^20–26^.

Although CAs are ubiquitous in nature, bacterial CAs from thermophiles, microorganisms living at temperatures ranging from 70 °C to 110 °C, are the mostly used ones as biocatalysts in CCP technology because of their ability to perform catalysis in the operatively hard conditions, typical of the industrial processes, such as extreme temperatures, pH values and in the presence of organic solvents^1^^,^^54^. In fact, it has been demonstrated that enzymes from thermophiles are thermostable, thermoactive up to 100 °C, and generally better supporting common enzyme denaturants^1^^,^^54^. Recently, our groups reported the discovery and characterisation of α-CAs from thermophilic bacteria, belonging to the genus *Sulfurihydrogenibium*, living in hot springs all over the world, at temperatures of up to 110 °C^55–57^. The α-CA identified in the bacterial species *S. yellowstonense* and indicated with the acronym (SspCA) retained its high catalytic activity for the CO_2_ hydration reaction even after being heated at 80 °C for several hours^46^^,^^56–59^. The α-CA, named SazCA and recognised in *S. azorense*, resulted the most active CA known to date, and the second most efficient enzyme (after superoxide dismutase), with a *k*_cat_ value of 4.40 × 10^6^ s^−1^ and a *k*_cat_/*K*_M_ value of 3.5 × 10^8^ M^−1 ^s^−143,^^55^^,^^60^^,^^61^. Moreover, our groups resolved the crystallographic structures of SspCa and SazCA in order to identify the molecular factors responsible for the higher thermostability of SspCA and the higher catalytic activity of SazCA with respect to the other bacterial and mammalian CAs known so far^43^^,^^46^.

Recently we reported a three-phase trickle-bed reactor containing the highly thermostable SspCA covalently immobilised within a polyurethane (PU) foam^1^. We demonstrated that when a gas phase containing CO_2_, was put in contact with the PU–SspCA suspended in a liquid phase, working in countercurrent, the CO_2_ was efficiently absorbed and converted into bicarbonate. Here, we described a heterologous expression of the recombinant SspCA carried out using high-density fermentation of *Escherichia coli* cultures, in order to produce SspCA which was covalently immobilised onto the surface of magnetic Fe_3_O_4_ nanoparticles (MNP) by using the carbodiimide activation reaction.

## Materials and methods

### Chemicals

The α-CA from the bovine erythrocytes (bCA) and all other chemicals used were commercial products of the purest quality and purchased from Sigma-Aldrich (Milan, Italy).

### Gene synthesis

The GeneArt Company (Invitrogen, Carlsbad, CA), specialised in gene synthesis, designed the synthetic *Sulfurihydrogenibium* sp. gene encoding for the SspCA lacking of the signal peptide (the first 20 amino acids at the N-terminal amino acid sequence) and containing four base-pair sequences (CACC) necessary for directional cloning at the 5′ end of the SspCA gene. The recovered SspCA gene and the linearised expression vector (pET-100/D-TOPO) were ligated by T4 DNA ligase to form the expression vector pET-100/SspCA.

### Large-scale production of the recombinant SspCA

BL21-CodonPlus (DE3)-RIPL competent cells (Agilent, Palo Alto, CA) were transformed with pET-100/SspCA and grown at 30 °C in a New Brunswick BioFlo 315 benchtop fermenter (Eppendorf, Hauppauge, NY) having the following configuration: air flow control, 2 L working volume vessel, direct drive motor, a water-temperature controller, a gas sparger, two peristaltic pumps to control pH by adding concentrate NaOH or HCL, and one peristaltic pump to control the foam by adding the antifoam 204 (Sigma-Aldrich, St. Louis, MO). As medium was used Terrific Broth (Sigma) to which has been added 150 ml 10× phosphate/citric acid buffer [133 g/L KH_2_PO_4_, 40 g/L (NH_4_)_2_HPO_4_ and 17 g/L citric acid]. Deionised water to a final volume of 2 L was added to the vessel before sterilisation at 121 °C for 20 min. After the solution was cooled to room temperature, the following sterile components were added to make the complete fermentation medium: 15 ml of 240 g/L MgSO_4_, 0.34 ml of 20 g/L thiamine, 15 ml of 100× trace element solution, and 22 ml of 70% glucose solution. The 100× trace element solution contained: 10 g/L iron (III) citrate, 0.25 g/L CoCl_2_·6H_2_O, 1.5 g/L MnCl_2_·4H_2_O, 0.15 g/L CuCl_2_·6H_2_O, 0.3 g/L H_3_BO_3_, 0.25 g/L Na_2_MoO_4_·2H_2_O, 1.3 g/L zinc acetate·2H_2_O, 0.84 g/L EDTA. The inoculum was grown in Terrific Broth medium. A 500 ml baffled shake flask containing 100 ml of TB medium were inoculated with 2–3 colonies of transformed *E. coli* and incubated at 30 °C, 200 rpm overnight in a New Brunswick Innova 40 benchtop incubator shaker (Eppendorf). After the overnight culture, the vessel was inoculated with 100 ml of inoculum (5% of the working vessel volume). *E. coli* cells were grown at 30 °C and induced with 1 mM IPTG. After 30 min, 0.5 mM ZnSO_4_ has been added. Cells were grown for additional 2 h and 20 ml of a feeding medium was added. The additional concentrate-feeding medium was prepared to mix 45 ml of 240 g/L MgSO_4_, 1.66 ml of 20 g/L thiamine solution, 15 ml of 100× trace element solution, and 70% glucose solution to a final volume of 500 ml. After the feeding, the culture medium and cells were grown for additional 2 h. Subsequently, cells were harvested and resuspended in the following buffer: 50 mM Tris/HCl, pH 8.0, 0.5 mM PMSF, and 1 mM benzamidine. Cells were then disrupted by sonication at 4 °C and centrifuged at 12,000*g* for 1 h. Following centrifugation, the cell extract was heated at 70 °C for 30 min and centrifuged at 12,000*g* for 30 min. At this stage of purification, the protein was at least 60% pure and the obtained recovery was of about 100 mg of the recombinant protein.

### Affinity chromatography

To obtain the pure SspCA, the supernatant was incubated with His Select HF nickel affinity gel resin (Sigma) equilibrated in lysis buffer for 30 min. Following centrifugation at 2000*g*, the resin was washed with wash buffer (50 mM Tris/HCl, pH 8.3, 500 mM KCl, 20 mM imidazole). The protein was eluted with the wash buffer containing 300 mM imidazole. Collected fractions were dialyzed against 50 mM Tris/HCl, pH 8.3. At this stage of purification, the amount of recovered SspCA was 90 mg and the protein was at least 90% pure.

### SDS–PAGE

Sodium dodecyl sulphate (SDS)–polyacrylamide gel electrophoresis (PAGE) was carried out according to Laemmli^62^. Samples were dissolved in buffer with 5% β-mercaptoethanol. The gel was stained with Coomassie blue. Protein concentration was determined by Bio-Rad assay kit (Hercules, CA).

### Carbonic anhydrase assay

Carbonic anhydrase activity assay was a medication of the procedure described by Capasso et al.^57^. Briefly, the assay was performed at 0 °C using CO_2_ as substrate following the pH variation due to the catalysed conversion of CO_2_ to bicarbonate. Bromothymol blue was used as the indicator of pH variation. The production of hydrogen ions during the CO_2_ hydration reaction lowers the pH of the solution until the colour transition point of the dye is reached. The time required for the colour change is inversely related to the quantity of CA present in the sample. Wilbur–Anderson units were calculated according to the following definition: One Wilbur–Anderson unit (WAU) of activity is defined as (T0 − T)/T, where T0 (uncatalysed reaction) and T (catalysed reaction) are recorded as the time (s) required for the pH to drop from 8.3 to the transition point of the dye in a control buffer and in the presence of enzyme, respectively. Generally, 100 mg of bound SspCA or bCA to the magnetic nanoparticles (MNP) were added to the test tube containing 1 ml of substrate solution. Free enzymes were assayed using in the test tube 10 ng of SspCA or bCA.

### Immobilisation of SspCA or bCA onto magnetic nanoparticles

Magnetic nanoparticles of Fe_3_O_4_ were prepared by coprecipitating Fe^2+^ and Fe^3+^ ions with aqueous ammonia solution. The ferric and ferrous chlorides (molar ratio of 2:1) were dissolved in water at a total concentration of 0.3 M iron. Chemical precipitation was achieved at 25 °C under vigorous stirring by adding the NH_4_OH solution (29.6%) and maintaining the pH at about 10. The precipitate was heated at 80 °C for 30 min, washed several times with a solution of water and ethanol (2:1), and finally dried in a vacuum oven at 70 °C. Magnetic particles (about 3 g) were stored at room temperature. For the binding of SspCA or bCA, 250 mg of MNP was first added to 2 ml of buffer 1 (0.003 M phosphate, pH 6.0, 0.1 M NaCl). Next, the reaction mixture was sonicated for 30 min after addition of 0.5 ml of carbodiimide solution (0.025 g/mL in buffer 1). Finally, 5 ml of SspCA or bCA solution at a concentration of 2 mg/mL in buffer 1 were added, and the reaction mixture was sonicated for 45 min. The binding process was carried out at a constant temperature of 4 °C. The SspCA or bCA bound to MNP were recovered from the reaction mixture by placing the bottle on an electromagnet with a surface of magnetisation of 10 cm. The magnetic particles settled within 5 min. The supernatant was used for protein analysis for determining the unbound proteins. The precipitates were washed with buffer 1 and then buffer 2 (0.1 M Tris, pH 8.0, containing 0.1 M NaCl for the flocculation of MNP) and then directly used for the measurements of activity and stability.

### Temperature studies

#### Effect of temperature on the free and immobilised SspCA and bCA

To compare the stability of SspCA and bCA free and immobilised at different temperatures, free enzymes at the concentration of 1 mg/ml in 10 mM Tris/HCl, pH 8.3 or MNP with the immobilised enzyme (1000 mg) were incubated at 50 and 70 °C for different time (6, 24 and 72 h). Free or bound enzymes aliquots were withdrawn at appropriate times and the residual activity was measured using CO_2_ as substrate. All data have been analysed by means of GraphPad Prism 5.0 software (GraphPad Software, San Diego, CA). Curves were obtained by the mean of three independent determinations.

#### Long-term stability

Free and bound CAs (SspCA and bCA) were examined for long-term stability (5, 15 and 30 days) at 25 °C by assaying their hydratase residual activities using CO_2_ as substrate. Free or bound enzymes aliquots were withdrawn at appropriate times for the measurements of the long-term enzyme stability. All the solutions containing the CAs were sterilised by using a sterile 0.22 µm PVDF filter and the aliquots were withdrawn under a sterile hood. All data were obtained by the mean of 3 independent determinations.

## Results and discussion

### SspCA production

**Figure 1.**
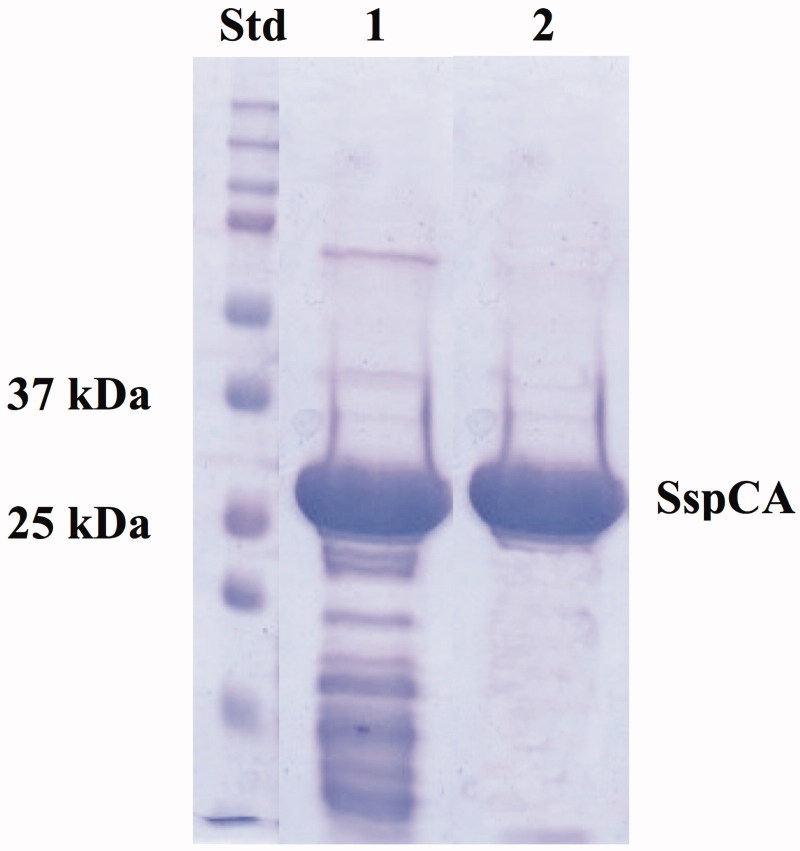
SDS–PAGE of the recombinant SspCA expressed and purified from *E. coli*. Lane STD, molecular markers, M.W. starting from the top: 250, 150, 100, 75, 50, 37, 25, 20 kDa; Lane 1, SspCA after thermoprecipitation at 70 °C and centrifugation; Lane 2, purified SspCA from His-tag affinity column.

The recombinant thermostable SspCA was heterologous expressed through a high cell density fermentation process using *E. coli* BL21-CodonPlus (DE3)-RIPL as host cells. The experiments were initially performed in shake flasks to formulate an alternative culture medium and to replace the medium in the large-scale experiments. The optimisation of the entire process using the bioprocess station, the selection of the expression vector, the choice of a particular bacterial strain, the use of a modified medium culture and the feeding strategy resulted in a significant enhancement of the biomass and protein production, compared to our previously reported procedure^1^. The biomass production was increased 20-fold in cell density with respect to the production usually carried out in our laboratories using 3 L flasks cultures. Consequently, a large amount of SspCA was detected in the soluble fraction after sonication and centrifugation of the cellular extract. Moreover, since the recombinant SspCA showed an extreme resistance toward high temperature^43^^,^^46^^,^^57^^,^^59–61^^,^^63^, the enzyme was partially purified by heating the bacterial extract at 70 °C for 30 min. After the thermoprecipitation, the specific activity of SspCA was recovered in the supernatant (Figure 1, lane 1), whereas most of the host proteins were thermo-precipitated and eliminated after centrifugation. At this step of purification and starting from about 30 g of bacterial biomass, SspCA was about 60% pure (Figure 1, lane 1) and its total protein amount was 100 mg, as estimated from the determined hydratase specific activity using CO_2_ as substrate. Moreover, the recombinant SspCA was expressed as a fusion protein with a His-Tag fragment containing six histidines at the N-terminal amino acid sequence of SspCA. Thus, using the affinity column (His-select HF Nickel affinity gel), SspCA was purified to an apparent homogeneity with a purity grade of about 90% (Figure 1, lane 2) as indicated by a single protein band after SDS–PAGE (Figure 1, lane 2). After the affinity chromatography, the total amount of pure SspCA was about 90 mg. Our results demonstrated that using a benchtop bioprocess station and the strategies aforementioned for optimising the bacterial growth, it was possible to produce “in house” and at low cost a rather high amount SspCA. Moreover, the thermoprecipitation is a cheap technique and a good strategy for obtaining enough SspCA to be used as biocatalyst in a bioreactor for the biomimetic CO_2_ capture.

### Immobilisation onto MNP

**Figure 2.**
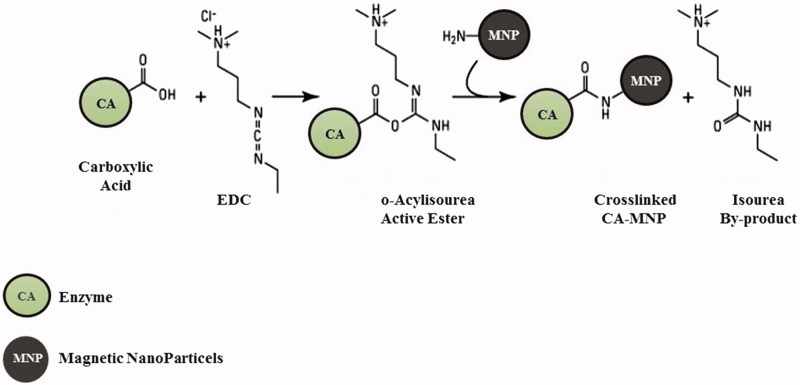
Schematic representation of the crosslinked carbonic anhydrase (CA) and magnetic nanoparticles (MNP) incorporating NH_2_ moieties obtained by reaction of hydrated magnetite with concentrated ammonia solution. The OH from magnetite may also be derivatised in a similar manner with formation of ester linkages.

**Figure 3.**
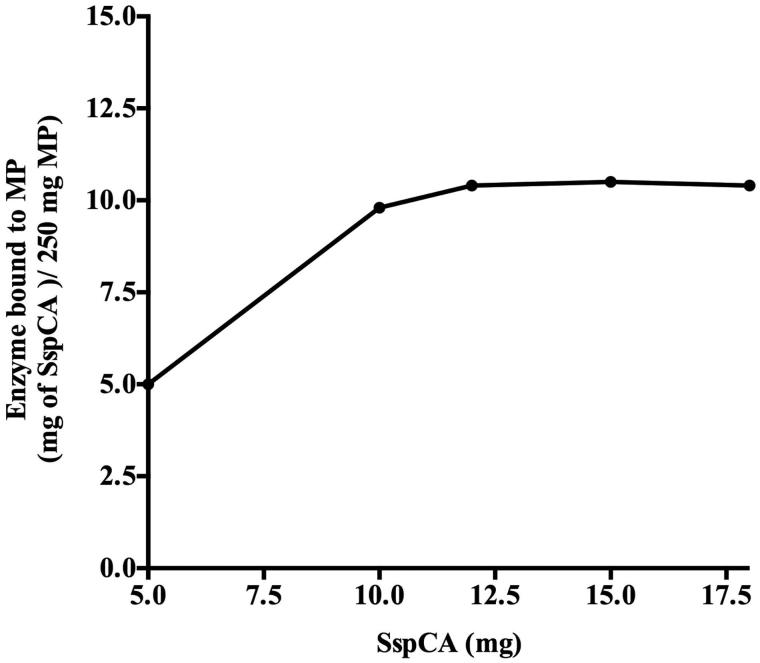
Binding of SspCA to MNPs. The amount of SspCA bound to 250 mg of Fe_3_O_4_ increased up to about 12 mg of enzyme. Measurements have been done determining the unbound SspCA in the supernatant after the binding process. Each point was the mean of three independent determinations. All data was analyzed by means of GraphPad Prism 5.0 software (GraphPad Software, San Diego, CA).

The improvement of the enzyme stability and the reuse of the enzyme are two important aspects for lowering the production costs of the biomimetic CCP. The enzyme stability is generally influenced by the industrial process conditions and this problem can be overcome by immobilising it on a specific support. The immobilisation improves the operational stability of the enzyme and avoids the diffusion of the macromolecules. In this context, we decided to immobilise SspCA and bCA (commercial bovine α-CA) onto MNP, which allows the separation of the CA from the reaction mixture and the reuse of the enzyme for many cycles. MNP can be easily captured using a magnetic field, such as a magnet or an electromagnet. SspCA (or bCA) was efficiently and directly bound to MNP prepared by coprecipitating Fe^2+^ and Fe^3+^ ions with aqueous ammonia solution. As reported in the literature, after the activation of the magnetite with carbodiimide (EDC) the binding was achieved via the reaction between the OH or NH_2_ groups present on the surface of hydrated magnetite nanoparticles (the last ones obtained from the first by reaction with the concentrated ammonia solution), and carboxyl groups of the thermostable SspCA or bCA^64^ (see Figure 2). By measuring the unbound protein in the supernatant after the binding process, the amount of SspCA or bCA bound to 250 mg of Fe_3_O_4_ increased up to about 12 mg of SspCA or bCA. As indicated in Figure 3, the amount of SspCA immobilised onto 250 mg of MNP was in the range of 10–12 mg of total enzyme (bCA showed a similar result). Moreover, measuring the hydratase activity using CO_2_ as substrate, we found that 100 mg of MNP with the immobilised enzyme showed a hydratase activity corresponding to that obtained using 100 ng of the unbound enzyme. These differences should be attributed to different phenomena, such as the reduced grade of the three-dimensional conformational changes of the immobilised enzyme, which is linked to the support matrix; the diverse microenvironment of the reaction between the substrate and the immobilised enzyme respect to those of the free enzyme; and the MNP aggregation during the assay.

### Effect of temperature and long-term stability of the free and immobilised SspCA and bCA

**Figure 4.**
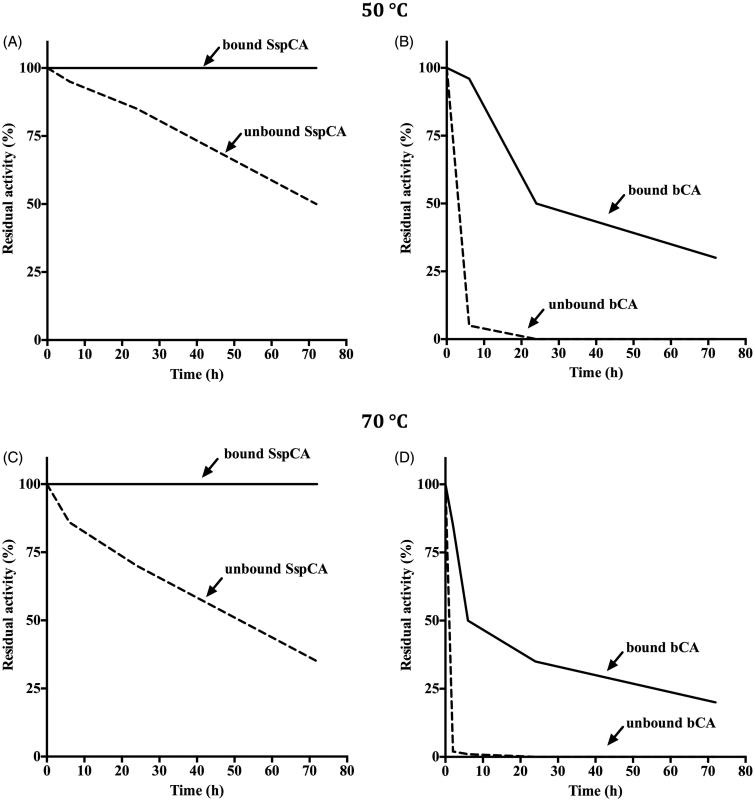
Temperature stability of the free and bound CAs (SspCA and bCA). Panel A and B: temperature stability at 50 °C; Panel C and D: temperature stability at 70 °C. Continuous line: bound SspCA or bCA; Dashed line: unbound SspCA or bCA. Each point is the mean of three independent determinations.

**Figure 5.**
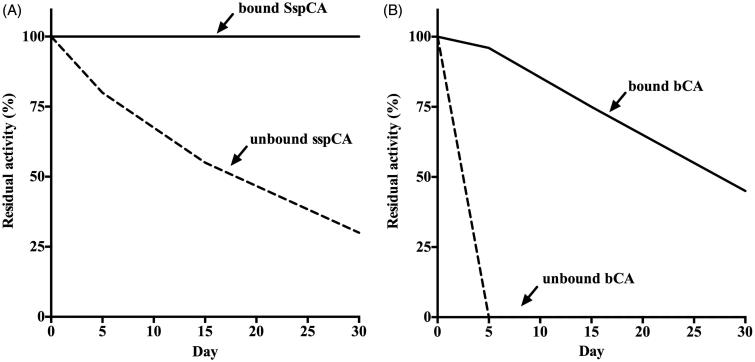
Long-term stability of the free and bound CAs (SspCA and bCA). Long-term stability was performed at 25 °C measuring the residual activity of the free and bound SspCA and bCA at the days indicated on the *x*-axis. Legend: Panel A: free and bound SspCA. Panel B: free and bound bCA. Continuous line: bound SspCA or bCA; Dashed line: unbound SspCA or bCA. Each point is the mean of three independent determinations.

The effect of temperature (at 50 and 70 °C) was determined for the free and bound CAs (SspCA and bCA), as shown in Figure 4. The residual activity of bound SspCA remained constant (100%) at both 50 and 70 °C for all times considered on the *x*-axis (Figure 4(A,C)). On the other hand, the immobilised mammalian enzyme (bCA) was less stable compared to the bound SspCA. In fact, in the first 10 h, the residual activity at 50 °C of the bound bCA was of 85% (Figure 4(B)), while at 70 °C decreased to 50% (Figure 4(B)). The unbound enzymes showed a different behaviour compared to the immobilised ones. In particular, by increasing the incubation time, the free SspCA residual activity at 50 °C decreased to about 50% and at 70 °C became of 30% after 70 h (Figure 4(A,C)). Instead, the free bCA lost most of its residual activity after 6 h at 50 °C, and after 3 h at 70 °C. These results demonstrated that the enzyme immobilisation onto MNP considerably increased the enzyme stability and the effect was more evident when the thermostable CA SspCA was immobilised. The bound enzymes continued to work at temperature considered prohibitive for free enzymes, such as 70 °C. We want to stress the fact that the temperature of the absorption column used for the biomimetic capture of CO_2_ typically ranges between 40 and 60 °C. Thus, the enzyme immobilisation is a good choice for enhancing the operational stability of the enzymes. Figure 5 shows the long-term stability of the free and bound SspCA and bCA at 25 °C and pH 8.3. After an incubation time of 30 days, the residual activity of the free SspCA was of 25% (Figure 5(A)). Interestingly, this value (25%) was reached by the free bCA in only 4 days (Figure 5(B)). In fact, free bCA completely lost its residual activity after 5 days. However, the bound SspCA retained 100% activity at 25 °C over a period of one month (Figure 5(A)), while the residual activity of the bound bCA was 50% after 30 days of incubation (Figure 5(B)). These results clearly demonstrate that the storage stability of the enzymes was significantly improved after binding to the MNPs.

## Conclusions

Using a benchtop bioprocess station and the strategies for optimising the bacterial growth, it was possible to produce “in house” and at low cost a rather high amount of SspCA. Moreover, the thermoprecipitation used in the purification process is a cheap technique and a good strategy for obtaining enough SspCA to be used as biocatalyst in a bioreactor for the biomimetic capture of CO_2_. It is possible to identify three major criteria that, in general, typify the immobilised enzymes: the easy separation of the enzyme from the product of the reaction; the increases in the enzyme stability by preventing the protein from unfolding to a certain degree; and the reuse of the enzyme. From our results, it is readily apparent that magnetic nanoparticle-immobilised CAs meets all three aforementioned criteria. In fact, the covalent immobilisation of SspCA directly onto the surface of magnetic Fe_3_O_4_ nanoparticles (MNP) via carbodiimide activation increased the stability and the log-term storage of the biocatalyst. Moreover, the bound SspCA to MNP can be recovered from the reaction mixture and reused simply applying a magnet or an electromagnet field because of the strong ferromagnetic properties of Fe_3_O_4_. All these aspects contribute to consider the thermostable SspCA a good candidate for the realisation of a bioreactor involved in the biomimetic capture of the CO_2_.
